# Evaluation of serum free fatty acids in chronic renal failure: evidence from a rare case with undetectable serum free fatty acids and population data

**DOI:** 10.1186/s12944-019-1093-5

**Published:** 2019-07-08

**Authors:** Zhen-Xian Liu, Qian Hong, Ding-Hui Peng, Ying Yang, Wen-Li Yu, Hua Shui, Xin Zhou, Song-Mei Liu

**Affiliations:** 1 Department of Clinical Laboratory, Center for Gene Diagnosis and Program of Clinical Laboratory, Zhongnan Hospital, Wuhan University, 169 Donghu Road, Wuhan, 430071 Hubei Province China; 2 Department of Nephrology, Renmin Hospital of Huangmei County, Huanggang, 435500 Hubei Province China; 3 Department of Nephrology, Zhongnan Hospital, Wuhan University, 169 Donghu Road, Wuhan, 430071 Hubei Province China

**Keywords:** Chronic renal failure, Free fatty acids, Proteinuria, Glucose metabolism, Lipid metabolism

## Abstract

**Background:**

Free fatty acid (FFA) accumulation in proximal tubules plays a fundamental role in the progress of kidney disease. Here, we reported a rare case with undetectable serum FFAs and further evaluated the changes of serum FFAs in patients with chronic renal failure (CRF).

**Methods:**

We analyzed the clinical data of a rare case and 574 CRF patients. The mRNA expression of lipoprotein lipase (LPL), hepatic lipase (HL) and fatty acid synthase (FASN) were determined in the rare case and 30 age-matched healthy males with qPCR.

**Results:**

This rare case had serious proteinuria, hyperglycemia, lipid disorders and bilateral renal glomerular filtration dysfunction. Compared with healthy males, this case showed a 1.49-fold increase of *LPL* expression (*P* < 0.01), a 3.38-fold reduction of *HL* expression (*P* < 0.001), and no significant change of *FASN* expression (*P* > 0.05). In total, 21.6% of CRF patients showed abnormal FFAs. Biochemical parameters such as blood urea nitrogen (BUN) and creatinine (CREA) significantly differed among groups with low-, normal- or high-level-FFAs. Moreover, serum FFAs was found to be associated with BUN. FFAs decreased in the group with higher BUN (> 17.4 mmol/L) and in the group with lower estimated glomerular filtration rate (eGFR) (< 15 mL/min/1.73m^2^).

**Conclusions:**

The proteinuria, *HL* low expression and renal function failure may contribute to the FFA reduction, which might imply that the renal function is severely damaged.

**Electronic supplementary material:**

The online version of this article (10.1186/s12944-019-1093-5) contains supplementary material, which is available to authorized users.

## Background

Chronic kidney disease (CKD) represents an ever-increasing worldwide health problem [1]. Hypertension, obesity, diabetes and chronic glomerular diseases are risk factors for CKD. Simultaneously, severe CKD could lead to cardiovascular diseases, fracture, anemia, hypertension, and other complications [2, 3]. Chronic renal failure (CRF) would occur if renal function failed to meet the body needs, requiring replacement therapy (i.e., renal dialysis or kidney transplantation) [4]. The causes of CKD differ by country, race and age. In the United States and the United Kingdom, end-stage kidney disease mainly results from diabetic nephropathy, while primary glomerulonephritis is the leading cause of CRF in China [5].

Free fatty acids (FFAs) are derived from triacylglycerol lipolysis governed by lipoprotein lipase (LPL) [6] and hepatic lipase (HL) [7], or de novo synthesis from acetyl-CoA, malonyl-CoA and NADPH by fatty acid synthases (FASN) [8]. In proximal tubules, FFAs act as energy sources, membrane components, and precursors of lipid mediators. FFAs could be filtered by glomeruli and reabsorbed into the proximal tubules through binding to albumin. In cases of severe proteinuria, hypoxia, or intoxication, FFAs would accumulate in proximal tubules [9]. Excessive FFAs could be esterified with glycerides and deposit in intracellular lipid droplets in the form of triglycerides, which might induce renal damage. In turn, renal dysfunction further aggravates the accumulation of FFAs in proximal tubules. FFA accumulation and overoxidation lead to podocytes structural damages, resulting in glomerulopathy and CRF [10]. Dyslipidemia may affect the kidney directly by lipotoxicity, as well as indirectly through inflammation, oxidative stress, vascular injury, and hormones changes [11]. With the development of CKD, dyslipoproteinemia becomes more pronounced and even could not be substantially improved by dialysis [12].

Abnormal FFA metabolism mediates the development of many diseases, including type 2 diabetes, cardiovascular diseases, and hypertension [13, 14], which are closely related to CRF [2, 3]. However, to date, only a few studies have focused on serum FFAs and CRF, and FFA profile has been only evaluated in patients undergoing dialysis or renal transplantation. Notably, very low serum FFAs are not common in clinical and laboratory observations.

Therefore, it is of great significance to reveal the changes of serum FFAs in the context of renal failure. In this study, we reported a rare case with undetectable serum FFAs and further evaluated the changes of serum FFAs in 574 CRF patients.

## Materials and methods

### Study subjects

A 43-year-old, male patient with undetectable serum FFAs and 574 CRF patients from May 2017 to August 2018 were recruited in this study. The average age for the 574 CRF patients was 62.9 ± 0.7 years and 64.1% (*n* = 368) were males. For genetic analysis of the case, 30 age-matched healthy males (43.9 ± 0.7 years) were also enrolled at Zhongnan Hospital of Wuhan University, China. All patients were diagnosed as CRF according to the Kidney Disease: Improving Global Outcomes (KDIGO) guidelines [15]. Healthy controls were randomly selected from physical examination population who had normal laboratory results, including liver function, kidney function, serum glucose, lipids and electrolytes, regular blood and urine tests. The exclusion criteria were diabetes, cardiovascular diseases or other serious diseases or use of any medication. This study was approved by the Ethics Committee of Zhongnan Hospital of Wuhan University and was performed according to the Declaration of Helsinki.

### Clinical biochemical tests

Biochemical parameters, including serum alanine transaminase (ALT), aspartate aminotransferase (AST), AST/ALT, total bilirubin (TBIL), direct bilirubin (DBIL), unconjugated bilirubin (UBIL), total protein (TP), albumin, globulin (GLB), gamma-glutamyl transpeptidase (GGT), alkaline phosphatase (ALP), total bile acid (TBA), superoxide dismutase (SOD), glucose (GLU), blood urea nitrogen (BUN), creatinine (CREA), uric acid, carbon dioxide (CO_2_), cystatin C, total cholesterol (TC), triglyceride (TG), high-density lipoprotein cholesterol (HDL-C), low-density lipoprotein cholesterol (LDL-C), apolipoprotein A1 (ApoA1), apolipoprotein B (ApoB), lipoprotein (a) (Lp(a)), FFA, phospholipid (PLIP), K^+^, Na^+^, Cl^−^, Ca^2+^, Mg^2+^, phosphate and the 24-h urinary total protein (24 h-TP) were assayed using AU5831 automated chemistry analyzer (Beckman, USA). Urine glucose and protein were determined by Urine Dry Chemical Analyzer (AX-4030, Japan).

### Calculation of eGFR

The estimated glomerular filtration rate (eGFR) was calculated according to Chronic Kidney Disease Epidemiology Collaboration creatinine equation: eGFR = 141 × min (Scr /κ,1)^α^ × max (Scr/κ, 1)^-1.209^ × 0.993^Age^ × (1.018 if female), (Scr, Serum creatinine; unit, mg/dL, 1 mg/dL = 88.4 μmol /L).Where κ = 0.7 for females or 0.9 for males; α = − 0.329 for females or − 0.411 for males [16].

### Total RNA extraction

Total RNA was isolated from fresh white blood cells from the case and 30 healthy males using Trizol Reagent (Invitrogen, USA) according to the manufacturer’s protocol.

### Gene expression determination

cDNA was synthesized using a reverse-transcription kit with DNase treatment (TOYOBO, Japan). mRNA expression of *LPL, HL, FASN* was evaluated in triplicates using the iTaq™ Universal SYBR GREEN Supermixes (BioRad, USA) on a CFX Connect™ Real-Time PCR Detection System (BioRad, USA) and double normalization to *β-ACTIN* and *GAPDH*. The primers are listed in Additional file 1: Table S1.

### Statistical analysis

**Fig. 1:**
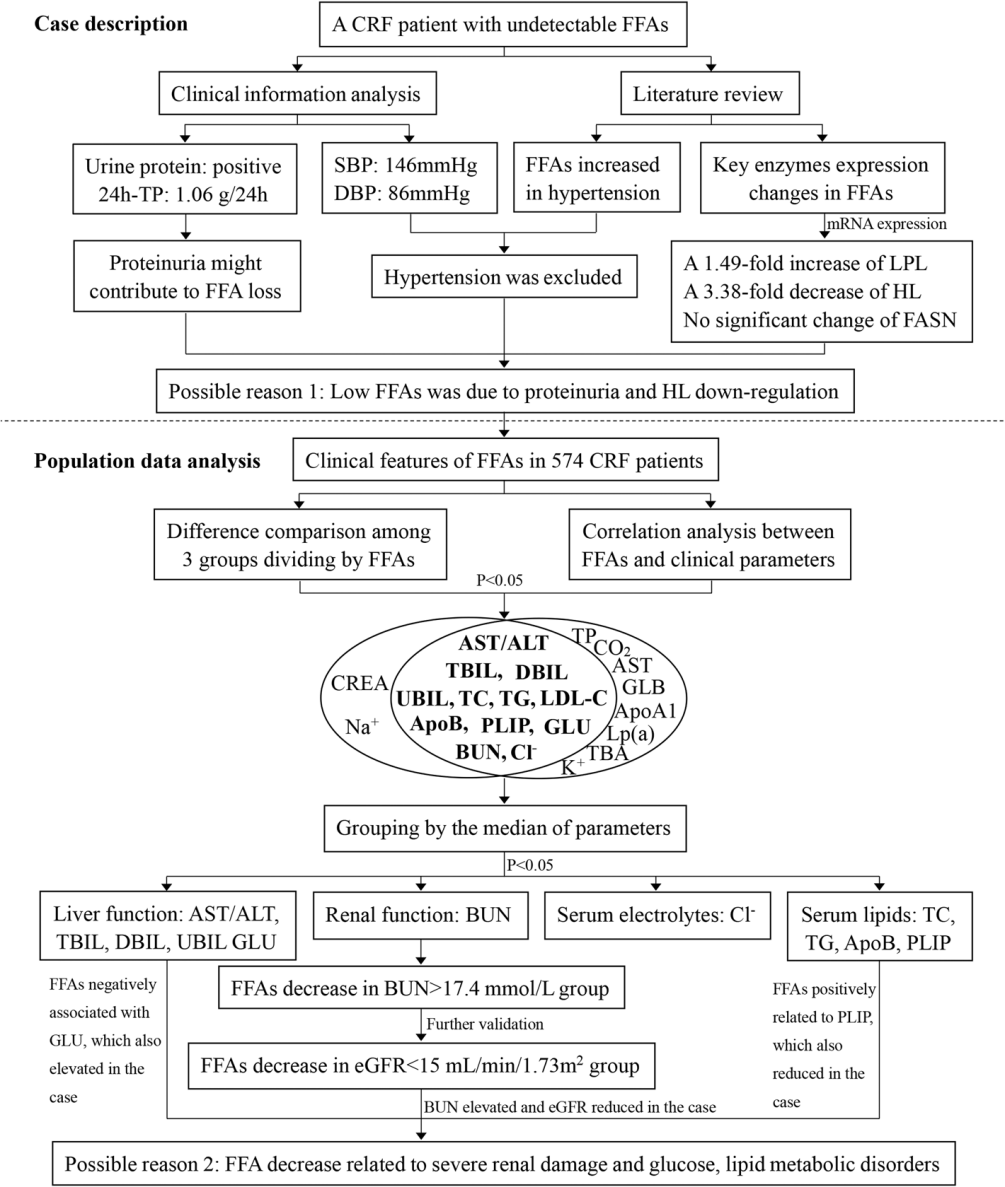
Study design. 24 h-TP, 24-h urinary total protein; SBP, Systolic Blood Pressure; DBP, Diastolic Blood Pressure; Bold fonts indicated that the parameters shown statistically significant in both difference comparison and correlation analysis

We used SPSS 20.0 to perform all statistical analyses. The flow chart of analysis is shown in Fig. 1. Continuous and normally distributed variables were presented as the mean ± standard deviation (M ± SD), and skewed variables were described by the median (interquartile range, IQR). The nonparametric Mann–Whitney U test was used to compare clinical parameters of CRF patients between different groups. The Spearman’s rank correlation test was used for correlation analysis. All statistical tests were two-sided, and *P*-value < 0.05 was considered statistically significant.

## Results

### Case description

A 43-year-old male was admitted to hospital because of CRF and persistently increased serum creatinine for 7 years, ranging from 451.0–930.8 μmol/L during his hospitalization. He had a history of hypertension for 7 years and took nifedipine controlled-release tablets, and his blood pressure was 146/86 mmHg on admission. He had no diabetes, heart disease, hepatitis B virus infection or tuberculosis, and never underwent hemodialysis. For CRF treatment, he also had calcium dobesilate to protect blood vessels and improve circulation, polysaccharide-Iron(III) complex to fight anemia, sodium bicarbonate to correct acidosis, calcium acetate tablets and vitamin D_3_ to keep balance of calcium and phosphate metabolism, as well as Hushen Keli, a traditional Chinese patent medicine to improve renal function.

As shown in Table 1, this patient had normal liver function, severely impaired renal function (i.e., increased serum BUN, CREA, Cystatin C, and decreased eGFR), hyperglycemia (GLU, 8.41 mmol/L), serum electrolyte disorders (i.e., decreased Cl^−^ and Ca^2+^, and increased Mg^2+^, phosphate). Interestingly, the case showed normal serum TC, TG, LDL-C, Lp(a), and decreased HDL-C, PLIP and undetectable FFAs. Additionally, urine glucose and protein were positive, and the 24 h-TP was 1.06 g/24 h (reference interval: 0–0.15 g/24 h). Further renal emission computed tomography (ECT) exam showed: (1) glomerular filtration rate (GFR): bilateral renal GFR was 16.0 mL/min (after correction, 16.4 mL/min/1.73m^2^), GFR for left and right single-kidney was 9.8 mL/min and 6.2 mL/min, respectively; (2) bilateral renal atrophy; (3) significantly reduction in bilateral renal blood perfusion; (4) severely impaired bilateral renal glomerular filtration function; (5) delayed bilateral renal excretion. The color Doppler ultrasound indicated that no obvious abnormality was observed in radial arteries of both upper limbs, cephalic veins, bilateral carotid or vertebral arteries. Based on the above mentioned results, the patient was diagnosed as CRF (CKD-5) and grade-3 hypertension (extremely high risk) with proteinuria, hyperglycemia and lipid disorders.

**Table 1:** Biochemical parameters of the CRF patient with undetectable serum FFAs

Parameter	Result	Reference interval
Liver function		
ALT (U/L)	11	9–50
AST (U/L)	**13**	15–40
AST / ALT	1.18	0.2–2
TBIL (μmol/L)	8.4	5–21
DBIL (μmol/L)	1.4	0–7
UBIL (μmol/L)	7	1.5–18
TP(g/L)	69	65–85
Albumin (g/L)	44.2	40–55
GLB (g/L)	24.8	20–30
Albumin/GLB	1.78	1.5–2.5
GGT(U/L)	29	8–57
ALP (U/L)	55	30–120
TBA (μmol/L)	3.4	0–15
SOD(U/L)	168.3	129–216
GLU (mmol/L)	**8.41**	3.9–6.1
Renal function		
BUN (mmol/L)	**46.4**	2.8–7.6
CREA (μmol/L)	**451**	64–104
Uric acid (μmol/L)	356.5	208–428
CO_2_ (mmol/L)	22.2	21–29
Cystatin C(mg/L)	**6.22**	0–1.2
eGFR (mL/min/1.73m^2^)	**12.8**	> 90
Serum lipids		
TC (mmol/L)	2.54	< 5.18
TG (mmol/L)	1.59	< 1.7
HDL-C(mmol/L)	**0.74**	> 1.04
LDL-C (mmol/L)	1.43	< 3.63
LP (a) (mg/L)	287.1	0–300
PLIP (mmol/L)	**1.61**	1.9–3.2
Serum electrolytes		
K^+^ (mmol/L)	3.95	3.5–5.3
Na^+^ (mmol/L)	138	137–147
Cl^−^ (mmol/L)	**97.2**	99–110
Ca^2+^(mmol/L)	**2.09**	2.11–2.52
Mg^2+^ (mmol/L)	**1.26**	0.85–1.15
Phosphate (mmol/L)	**2.14**	0.85–1.51

*ALT* alanine transaminase, *AST* aspartate aminotransferase, *TBIL* total bilirubin, *DBIL* direct bilirubin, *UBIL* unconjugated bilirubin, *TP* total protein, *GLB* globulin, *GGT* gamma-glutamyl transpeptidase, *ALP* alkaline phosphatase, *TBA* total bile acid, *SOD* superoxide dismutase, *GLU* glucose, *BUN* blood urea nitrogen, *CREA* creatinine, *CO*_*2*_ carbon dioxide, *eGFR* estimated glomerular filtration rate, *TC* total cholesterol, *TG* triglyceride, *HDL-C* high-density lipoprotein cholesterol, *LDL-C* low-density lipoprotein cholesterol, *Lp(a)* lipoprotein (a), *PLIP* phospholipid. Bold fonts indicated the results are abnormal

### Serum FFAs analysis of the case and literature review

To confirm that the case indeed had undetectable FFAs, we tested his serum FFAs and commercial FFA quality controls for 3 times. The test results showed that quality controls had expected values, and serum FFAs of the patient was only 0.07 μmol/L at the 2^nd^ run detection. Next, to avoid that serum FFA concentration was too high to detect, we tested FFAs with diluted serum, and the results were similar to the undiluted serum. Thus, we attempted to interpret this interesting clinical observation.

In general, FFAs are mainly contained in triglyceride-riched lipoproteins or bind to albumin in blood circulation [17]. Albumin-bound FFAs could be filtered through the glomeruli and accumulate in the proximal tubules in the setting of massive proteinuria [9]. Given this case had serious proteinuria (24 h-TP, 1.06 g/24 h; urine protein, positive), along with badly impaired bilateral renal GFR, one possible reason for the undetectable FFAs should be FFAs lost with urine protein.

**Fig. 2:**
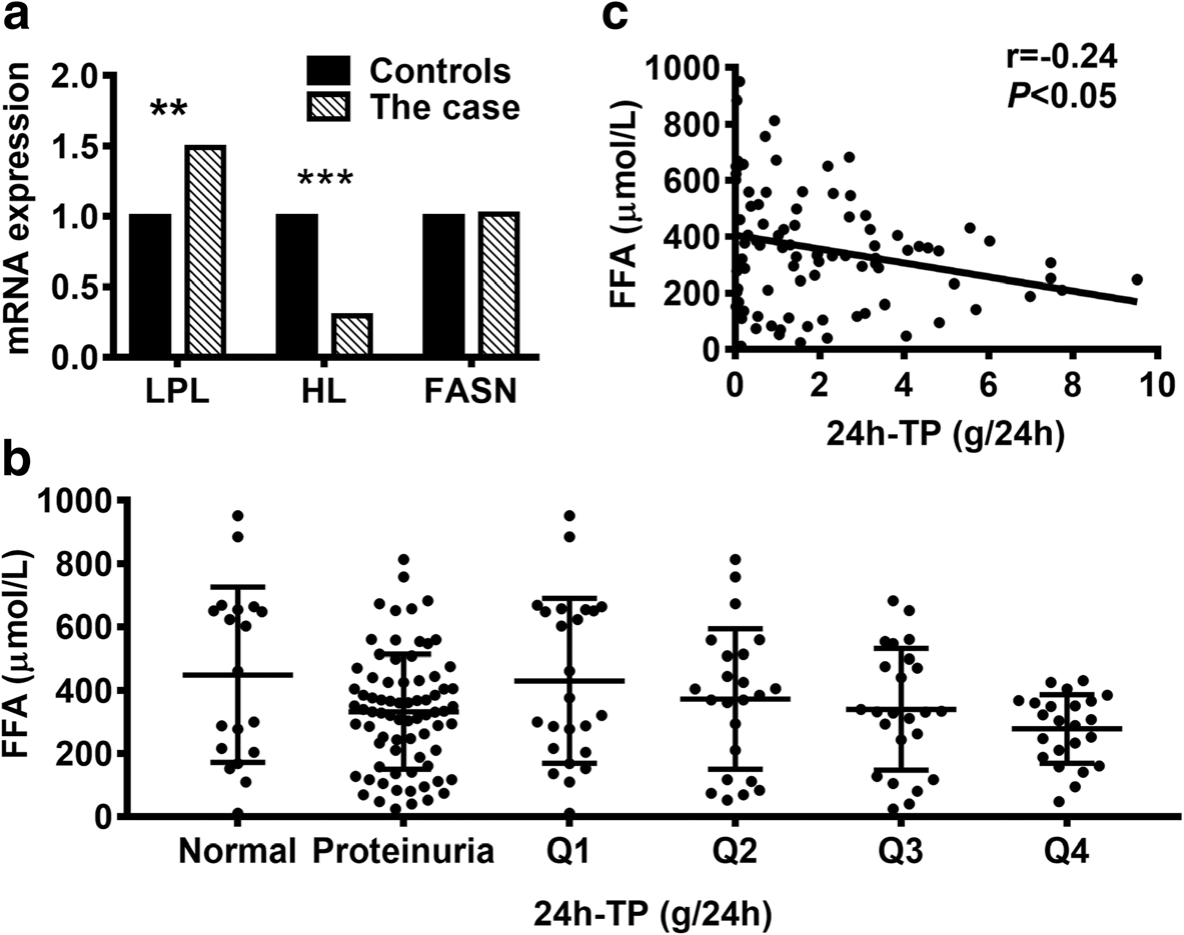
Analysis of gene expression and the 24-h urinary total proteins. **a** The mRNA expression of three key enzymes in FFA metabolism. Compared with 30 age-matched healthy males, *LPL* expression increased 1.49-fold but *HL* expression decreased 3.38-fold. *FASN* expression had no significant change. **b** Serum FFAs decreased in patients with proteinuria, and showed a decrease tendency with proteinuria aggravating. **c** Serum FFAs have a negative correlation with 24 h-TP. **P < 0.01, ***P < 0.001, Q1–4 indicated the quartile of 24 h-TP. FFA, free fatty acids; LPL, lipoprotein lipase; HL, hepatic lipase; FASN, fatty acid synthase; 24 h-TP, 24-h urinary total protein

Fig 2a shows the mRNA levels of *LPL, HL* and *FASN.* Compared with healthy males, the case had a 1.49-fold increase of *LPL* expression (*P* < 0.01), a 3.38-fold reduction of *HL* expression (*P* < 0.001), and no significant change of *FASN* expression (*P* > 0.05). It appeared that the fold-change of *HL* expression was greater than that of *LPL*. The increased *LPL* expression might not be able to compensate for the alteration triggered by *HL* low expression. Thus, the significant down-regulation of *HL* might be also responsible for the undetectable FFAs.

Hypertension and CRF have found to be the cause and effect in FFA metabolic disorders [2, 14, 18]. FFAs increased in patients with hypertension, suggesting that a higher level of FFAs was an independent risk factor for hypertension [14, 19]. To our surprise, the case had severe hypertension (grade-3) but with undetectable FFAs. The discrepancy between the case and previous studies might imply that FFA metabolism was complicated in CRF circumstance, and some unknown factors might also have contributed to the undetectable FFAs.

Taken together, the undetectable FFA in this CRF patient was mainly caused by FFA elimination with urine protein and down-regulation of *HL*.

### Population data analysis

To provide further evidence for the undetectable FFAs in this case and understand FFA changes in CRF patients, we systematically evaluated the alterations of FFAs and clinical parameters in 574 CRF patients. Of them, 13.59% had decreased FFAs, 8.01% had increased FFAs, and 78.4% were normal.

Based on the reference interval of serum FFAs (129–769 μmol/L), we divided the 574 participants into three groups: FFA-reduced (< 129 μmol/L), FFA-normal, and FFA-increased (> 769 μmol/L). The average of FFAs in the FFA-reduced group was 82.16 μmol/L, and the lowest value was 11.28 μmol/L. Next, we examined the difference of laboratory indicators among the three groups with K-independent nonparametric test, including liver function, kidney function, serum lipids and electrolytes (Table 2). No significant difference was found in age (*P* = 0.053) or gender (*P* = 0.072) distribution. Serum AST/ALT, TBIL, DBIL, UBIL, GLU, BUN, CREA, TC, TG, LDL-C, ApoB, PLIP, Na^+^ and Cl^−^ significantly differed across the three groups. For instance, liver function parameters (i.e., AST/ALT, TBIL, DBIL and UBIL) showed an increasing trend with FFA increasing (*P* < 0.01); serum GLU elevated in both the FFA-reduced group and the FFA-increased group (*P* < 0.01) when compared to the FFA-normal group. As for renal function indicators, serum BUN in the FFA-reduced group was the highest among the three groups (*P* < 0.01); serum CREA was apparently higher than that in the FFA-normal group (*P* < 0.01). Serum lipids differed across the three groups, except for HDL-C, ApoA1 and Lp(a). Also, serum Na^+^ and Cl^−^ showed statistically difference across the three groups. Therefore, our data provided strong evidence that serum FFAs were closely related to the renal function, given the long-standing recommendations for clinical evaluation of renal function with serum BUN and CREA.

**Table 2:** The differences of clinical parameters across three groups dividing by serum FFAs

Parameter	FFA-reduced group (*n* = 78)	FFA-normal group (*n* = 450)	FFA-increased group (*n* = 46)	*P*-value
Age (year)	59.3± 1.9	66.0(52.0–76.0)	66.4 ± 2.2	0.053
Liver function				
ALT (U/L)	16.50(10.00–25.25)	13.00(8.00–20.00)	12.00(7.75–19.25)	0.091
AST (U/L)	18.00(12.75–25.25)	18.00(14.00–25.00)	22.00(15.5–33.25)	0.078
AST/ALT	1.17(0.81–1.68)	1.38(1.00–1.75)	1.87(1.00–2.86)	0.001
TBIL (μmol/L)	8.15(6.70–10.48)	8.50(7.10–11.30)	10.45(8.50–15.65)	< 0.001
DBIL (μmol/L)	1.35(1.00–2.10)	1.50(1.10–2.20)	2.25(1.58–4.15)	< 0.001
UBIL (μmol/L)	6.80(5.70–8.53)	7.00(5.83–8.90)	8.05(6.93–12.15)	0.004
TP (g/L)	62.25(59.70–68.60)	64.50(58.90–70.10)	65.58 ± 1.12	0.169
Albumin (g/L)	33.94 ± 0.63	34.80(31.20–39.08)	34.77 ± 0.90	0.599
GLB (g/L)	28.81 ± 0.62	29.20(26.20–33.48)	30.81 ± 0.85	0.110
Albumin / GLB	1.14(1.04–1.44)	1.19(1.00–1.37)	1.18 ± 0.05	0.791
GGT (U/L)	24.50(15.00–50.00)	24.00(16.00–43.50)	26.50(17.00–60.00)	0.699
ALP (U/L)	83.50(63.50–96.25)	86.00(68.00–108.00)	88.00(63.75–110.00)	0.480
TBA (μmol/L)	4.35(2.68–6.63)	3.80(2.10–6.50)	3.50(1.78–8.90)	0.350
SOD (U/L)	135.54 ± 4.34	127.25 ± 1.88	136.35 ± 4.78	0.200
GLU(mmol/L)	6.56(5.25–7.99)	5.05(4.50–6.47)	6.62(4.87–9.37)	< 0.001
Renal function				
BUN (mmol/L)	21.60(15.10–26.05)	16.75(11.30–23.25)	15.65(10.55–22.83)	0.002
CREA (μmol/L)	688.69 ± 44.73	479.50(253.60–765.00)	593.93 ± 61.47	0.005
Uric acid (μmol/L)	438.17 ± 16.93	417.80(333.98–529.58)	443.67 ± 26.47	0.778
CO_2_ (mmol/L)	21.45 ± 0.62	22.39 ± 0.22	22.10 ± 0.74	0.419
Cystatin C (mg/L)	4.08(3.17–5.24)	3.78(2.85–5.31)	4.31 ± 0.36	0.258
eGFR (mL/min/1.73m^2^)	7.13(4.63–15.01)	8.94(5.36–18.45)	7.10(5.29–22.04)	0.147
Serum lipids				
TC (mmol/L)	3.55 ± 0.12	3.80(3.20–4.68)	3.70(3.25–4.85)	0.009
TG (mmol/L)	1.56(0.79–1.73)	1.38(1.01–2.04)	1.99(1.39–2.58)	< 0.001
HDL-C (mmol/L)	0.93 ± 0.03	0.90(0.72–1.11)	0.84 ± 0.06	0.088
LDL-C (mmol/L)	1.86 ± 0.07	1.97(1.62–2.62)	2.15 ± 0.12	0.009
ApoA1 (g/L)	0.99 ± 0.04	1.00(0.84–1.19)	0.95 ± 0.08	0.243
ApoB (g/L)	0.58 ± 0.03	0.67(0.55–0.80)	0.72 ± 0.05	0.018
Lp(a) (mg/L)	171.35(79.48–349.40)	152.35(73.25–316.03)	125.45(63.48–340.75)	0.420
FFA (μmol/L)	85.90(55.43–107.07)	348.31(247.31–480.56)	964.61(848.95–1283.06)	< 0.001
PLIP (mmol/L)	1.96 ± 0.59	2.06(1.82–2.36)	2.10(1.82–2.67)	0.017
Serum electrolytes				
K^+^ (mmol/L)	4.47 ± 0.10	4.32(3.90–4.91)	4.25 ± 0.14	0.178
Na^+^ (mmol/L)	138.20(135.50–141.03)	138.75(136.50–140.80)	136.75(133.93–139.78)	0.020
Cl^−^ (mmol/L)	104.20(101.53–109.15)	104.64 ± 0.27	100.72 ± 0.85	< 0.001
Ca^2+^ (mmol/L)	2.08 ± 0.03	2.17(2.00–2.32)	2.18 ± 0.05	0.050
Mg^2+^ (mmol/L)	0.96 ± 0.02	0.95(0.85–1.05)	0.97 ± 0.04	0.939
Phosphate (mmol/L)	1.53(1.23–1.96)	1.46(1.15–1.91)	1.56(0.98–1.96)	0.454

*ApoA1* apolipoprotein A1, *ApoB* apolipoprotein B, *FFA* free fatty acid

In addition to the BUN and CREA, the urine 24 h-TP is another important indicator for renal function. We therefore evaluated serum FFAs in 98 CRF patients who had results of urine 24 h-TP. Of the 98 patients, 19 patients had normal 24 h-TP (≤ 0.15 g/24 h) and 79 patients showed abnormal increased 24 h-TP. The median of 24 h-TP in this cohort was 1.395 g/24 h (IQR, 0.280–3.115). Consequently, CRF patients with higher urine 24 h-TP were more likely to have lower concentrations of FFAs (Fig. 2b), regardless of grouping by reference interval or the IQR range. More importantly, serum FFAs was found to be negatively associated with urine 24 h-TP (r = − 0.24, *P* < 0.05, Fig. 2c).

Furthermore, the Spearman’s rank correlation analysis revealed that FFAs had positive correlations with AST, AST/ALT, TBIL, DBIL, UBIL, TP, GLB, CO_2_, TC, TG, LDL-C, ApoB, PLIP, and negative correlations with TBA, GLU, BUN, ApoA1, Lp(a), K^+^ and Cl^−^ (Table 3).

**Table 3:** The correlations between serum FFAs and clinical parameters

Parameter	r	*P*-value	Number of Cases
Liver function			
ALT	−0.056	0.181	572
AST	0.089	0.033	573
AST/ALT	0.138	0.001	568
TBIL	0.275	< 0.0001	566
DBIL	0.230	< 0.0001	566
UBIL	0.259	< 0.0001	568
TP	0.127	0.002	569
Albumin	0.068	0.104	572
GLB	0.102	0.014	572
Albumin/GLB	−0.037	0.378	572
GGT	0.046	0.269	572
ALP	0.014	0.741	572
TBA	−0.13	0.002	572
SOD	0.038	0.445	401
GLU	−0.137	0.001	553
Renal function			
BUN	−0.135	0.001	568
CREA	−0.069	0.097	574
Uric acid	0.052	0.212	574
CO2	0.102	0.015	562
Cystatin C	−0.082	0.072	476
eGFR	0.042	0.317	558
Serum lipids			
TC	0.141	0.001	574
TG	0.225	< 0.0001	574
HDL-C	−0.038	0.364	574
LDL-C	0.109	0.009	574
ApoA1	−0.118	0.040	301
ApoB	0.125	0.029	307
LP (a)	−0.099	0.018	566
PLIP	0.175	0.0002	458
Serum electrolytes			
K^+^	−0.115	0.006	567
Na^+^	−0.058	0.170	571
Cl^−^	−0.192	< 0.0001	569
Ca^2+^	0.081	0.053	572
Mg^2+^	0.019	0.701	410
Phosphate	−0.034	0.426	567

**Fig. 3:**
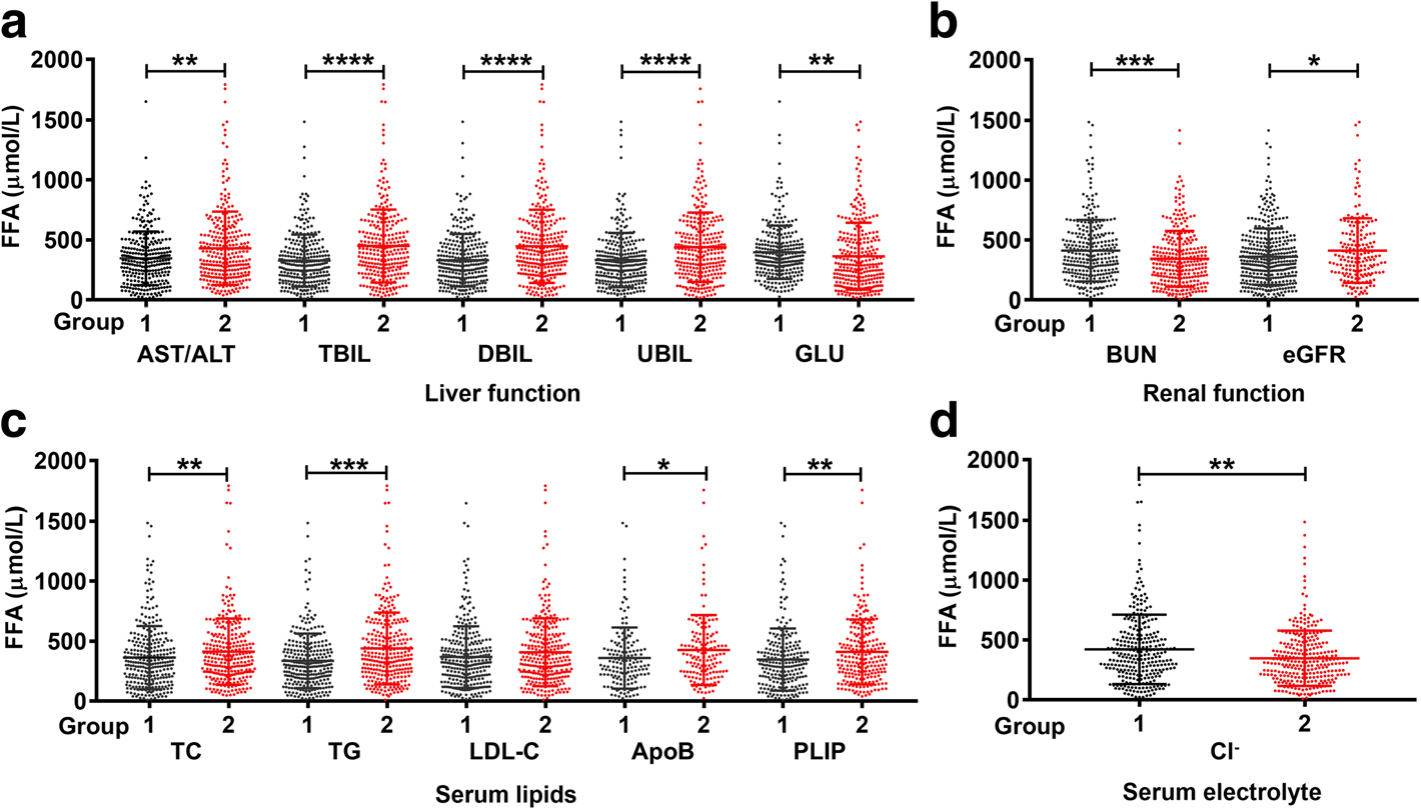
The differences of serum FFAs between groups. **a** Liver function. **b** Renal function. **c** Serum lipids. **d** Serum electrolytes. Group-1 ≤ the median and Group-2 > the median. The median of each parameter was: AST/ALT, 1.38; TBIL, 8.6 μmol/L; DBIL, 1.5 μmol/L; UBIL, 7.05 μmol/L; GLU, 5.31 mmol/L; BUN, 17.4 mmol/L; TC, 3.75 mmol/L; TG, 1.41 mmol/L; LDL-C, 1.96 mmol/L; ApoB, 0.66 g/L; PLIP, 2.04 mmol/L; Cl^−^, 104.25 mmol/L. The cutoff value for eGFR grouping is 15 mL/min/1.73m^2^. *P < 0.05, **P < 0.01 ***P < 0.001, ****P < 0.0001. ALT, alanine transaminase; AST, aspartate aminotransferase; TBIL, total bilirubin; DBIL, direct bilirubin; UBIL, unconjugated bilirubin; GLU, glucose; BUN, blood urea nitrogen; eGFR, estimated glomerular filtration rate; TC, total cholesterol; TG, triglyceride; LDL-C, low-density lipoprotein cholesterol; ApoB, apolipoprotein B; PLIP, phospholipid

As shown in Fig. 1, serum AST/ALT, TBIL, DBIL, UBIL, GLU, BUN, TC, TG, LDL-C, ApoB, PLIP, Cl^−^ not only differed significantly (all *P* < 0.05) across the three groups, but also were significantly associated with serum FFAs. Therefore, we divided the patients into group-1 (≤ median) and group-2 (> median) on the basis of the median of the above clinical parameters to assess serum FFAs. As illustrated in Fig. 3, the Mann–Whitney U test revealed that serum FFAs were significantly increased in group-2 (AST/ALT, TBIL, DBIL, UBIL, TC, TG, ApoB, PLIP) and in group-1 (GLU, BUN, Cl^−^). In agreement with that BUN was an important biomarker for renal function evaluation [20], of the 11 indicators, the abnormal rate of BUN was approximately 92.29%, while the rest was less than 50% (Additional file 1: Table S2). Particularly, the inverse correlation between FFAs and BUN further implied that the lower FFAs reflected poorer renal function.

Additionally, the eGFR was used to evaluate renal function for the vast majority of CKD patients in clinical practice due to convenience and cost-efficiency. The KDIGO guidelines also recommend that CRF patients with eGFR< 15 mL/min/1.73m^2^ should be classified as CKD-5 stage [15]. We therefore divided patients into two groups by eGFR: group-1 (eGFR < 15 mL/min/1.73m^2^) and group-2 (eGFR-2 ≥ 15 mL/min/1.73m^2^). Fig. 3b demonstrates that FFAs increased in the group-1, indicating inverse correlation between FFAs and kidney function. Consistent with the population data, the case with undetectable FFAs also showed elevated GLU, BUN, and decreased eGFR, PLIP. Collectively, our data demonstrated that FFA reduction was strongly associated with glucose, lipid metabolic disorders, and severe renal damages.

## Discussion

In the current study, we found that a rare case with undetectable FFAs had severe renal dysfunction, hypertension, hyperglycemia, proteinuria and *HL* down-regulation. Further population data analysis demonstrated that 21.6% CRF patients had abnormal serum FFAs. Specifically, more CRF patients had decreased FFAs when compared to patients with increased FFAs (13.6% vs. 8.0%). FFA reduction could be caused by multiple factors, including loss with proteinuria, hyperglycemia, lipid metabolic disorders, severely impaired renal function and dysregulation of FFA-related enzymes.

In line with a previous report that FFAs showed renoprotective potential, renal transplant recipients with higher plasma FFAs had lower risk of graft failure [21], our population data also suggested that lower serum FFAs indicated worse kidney function in CRF patients. The care with undetectable FFAs had dramatically reduced eGFR (12 mL/min/1.73m^2^), proteinuria, and significantly increased BUN, CREA. By telephone follow-up, we noticed that this patient had been taking hemodialysis owing to out of controlled serum CREA (> 1000 μmol/L) for 4 months after he left hospital.

Notably, different individual FFAs had various effects on renal function. In most cells such as podocytes, the overload of saturated fatty acid could induce insulin resistance and cell death via lipid toxicity, whereas monounsaturated fatty acid could reverse this lipotoxicity [22]. Older individuals with a higher level of plasma polyunsaturated fatty acids were at lower risk of developing renal insufficiency [23]. CKD patients under hemodialytic treatment had an increase of monounsaturated fatty acids and a decrease of n-3 polyunsaturated fatty acids [18]. Earlier studies also observed that polyunsaturated fatty acids intake showed protective effects on renal function [3, 24].

Hyperglycemia was a risk factor for CKD [25]. In this study, serum GLU abnormally elevated in the rare case, similar results were also found in CRF patients with increased or reduced FFAs. Additionally, CRF patients with higher serum GLU and lower serum FFAs exhibited apparent reduction of eGFR (Additional file 1: Figure S1). Our data implied that, to some extent, serum lower FFAs and hyperglycemia promoted renal dysfunction in CRF patients.

Besides, the relation of population data between FFAs with AST/ALT, TBIL, DBIL and UBIL suggested normal liver function was vital for FFA metabolism. Serum FFAs depended on the balance between FFA release from adipose tissue and FFA uptake/oxidation by liver and muscle tissues [26]. In our study, FFA alterations were closely related to serum levels of TC, TG, LDL-C and ApoB, which were consistent with the source and metabolism of FFAs. In terms of serum electrolytes, we only found that Cl^−^ was inversely correlated with FFAs. Interestingly, a recent study has proved that elevated FFAs and triacylglycerol levels directly reduced blood Mg^2+^ levels in metabolic disorders [27].

Nevertheless, this study could not rule out the limitations and challenges. Firstly, we only determined the mRNA expression of *LPL*, *HL* and *FASN* in the case and 30 healthy males, these results should be further confirmed in a larger sample size study and covered more genes related to FFA metabolism. Secondly, owing to treatment requirements, the patients were not under standard diet, exercise and same medications, we therefore were unable to adjust for these factors in the statistical analyses. It is not surprising that all these limitations did not pose a serious problem to our findings that low FFAs indicated severe renal damages based on the large population data analysis.

## Conclusions

Through clinical data analysis, literature review and gene expression of FFA-related metabolic enzymes, we concluded that the case with undetectable FFAs might be caused by proteinuria, *HL* low expression, glucose and lipid metabolism disorders, and severe renal failure. More importantly, our study revealed the associations between serum FFAs and renal dysfunction, and highlighted the great clinical significance of monitoring serum lipids, especially FFAs, for CRF patients.

## Additional file

Additional file 1: **Table S1.** Primers used in this study for qPCR. **Table S2.** The distributions of clinical parameters showing significant effects on serum FFAs. **Figure S1.** Serum FFAs and GLU exerted an opposite effect on renal function in CRF patients. (DOCX 105 kb)

## Data Availability

The datasets used and analyzed during the current study are available from the corresponding author on reasonable request.

## Abbreviations

24 h-TP: 24-h urinary total protein
ALP: Alkaline phosphatase
ALT: Alanine transaminase
ApoA1: Apolipoprotein A1
ApoB: Apolipoprotein B
AST: Aspartate aminotransferase
BUN: Blood urea nitrogen
CKD: Chronic kidney disease
CO_2_: Carbon dioxide
CREA: Creatinine
CRF: Chronic renal failure
DBIL: Direct bilirubin
DBP: Diastolic blood pressure
eGFR: Estimated glomerular filtration rate
FASN: Fatty acid synthase
FFAs: Free fatty acids
GFR: Glomerular filtration rate
GGT: Gamma-glutamyl transpeptidase
GLB: Globulin
GLU: Glucose
HDL-C: High-density lipoprotein cholesterol
HL: Hepatic lipase
IQR: Interquartile range
KDIGO: Kidney Disease: Improving Global Outcomes
LDL-C: Low-density lipoprotein cholesterol
Lp(a): Lipoprotein (a)
LPL: Lipoprotein lipase
PLIP: Phospholipid
SBP: Systolic Blood Pressure
Scr: Serum creatinine
SD: Standard deviations
SOD: Superoxide dismutase
TBA: Total bile acid
TBIL: Total bilirubin
TC: Total cholesterol
TG: Triglyceride
TP: Total protein
UBIL: Unconjugated bilirubin
