# Supplementary material for: Evaluation of serum free fatty acids in chronic renal failure: evidence from a rare case with undetectable serum free fatty acids and population data
Source: Lipids Health Dis. 2019 Jul 8;18:151. doi: 10.1186/s12944-019-1093-5 (PMC6615299; doi:10.1186/s12944-019-1093-5)
Supplement: Supplementary file 1 — Table S1. Primers used in this study for qPCR. Table S2. The distributions of clinical parameters showing significant effects on serum FFAs. Figure S1. Serum FFAs and GLU exerted an opposite effect on renal function in CRF patients. (DOCX 105 kb) [file 12944_2019_1093_MOESM1_ESM.docx]

**Evaluation of serum free fatty acids in chronic renal failure: evidence from a rare case with undetectable serum free fatty acids and population data**

Zhen-Xian Liu^1,†^, Qian Hong^2,3†^, Ding-Hui Peng^1,†^, Ying Yang^1^, Wen-Li Yu^3^, Hua Shui^3^, Xin Zhou^1^, Song-Mei Liu^1,*^

^1^ Department of Clinical Laboratory, Center for Gene Diagnosis & Program of Clinical Laboratory, Zhongnan Hospital of Wuhan University, Donghu Road 169#, Wuhan, 430071, China.

^2^ Department of Nephrology, Renmin Hospital of Huangmei County, Huanggang, Hubei, 435500, China.

^3^ Department of Nephrology, Zhongnan Hospital of Wuhan University, Donghu Road 169#, Wuhan, 430071, China.

Zhen-Xian Liu, [2015467359@qq.com](mailto:2015467359@qq.com)

Qian Hong, [530610003@qq.com](mailto:530610003@qq.com)

Ding-Hui Peng, [pengstm@163.com](mailto:pengstm@163.com)

Ying Yang, [yangying0109@whu.edu.cn](mailto:yangying0109@whu.edu.cn)

Wen-Li Yu, [ywlnw@sina.com](mailto:ywlnw@sina.com)

Hua Shui, [shuihua@whu.edu.cn](mailto:shuihua@whu.edu.cn)

Xin Zhou, [115634093@qq.com](mailto:115634093@qq.com)

†These authors contributed equally to this work.

*To whom correspondence should be addressed. Tel. +86-27-67813233; fax. +86-27-67813233. E-mail address: Song-Mei Liu, smliu@whu.edu.cn.

Table S1. Primers used in this study for qPCR.

| **Gene** | **Forward** | **Reverse** |
| --- | --- | --- |
| LPL | TCATTCCCGGAGTAGCAGAGT | GGCCACAAGTTTTGGCACC |
| HL | GGAACGCACAAGATTGGGAGAA | CCGTTGGGATAGAAGTCATAGTGTC |
| FASN | AGAACTTGCAGGAGTTCTGGGACA | TCCGAAGAAGGAGGCATCAAACCT |
| GAPDH | ATGACATCAAGAAGGTGGTG | CATACCAGGAAATGAGCTTG |
| β-actin | CCTGGCACCCAGCACAAT | GGGCCGGACTCGTCATACT |

LPL, lipoprotein lipase; HL, hepatic lipase; FASN, fatty acid synthase.

Table S2. The distributions of clinical parameters showing significant effects on serum FFAs.

| **Parameter** | **FFA-reduced group**  **(N;%)** | **FFA-normal group**  **(N;%)** | **FFA-increased group**  **(N;%)** |
| --- | --- | --- | --- |
| AST/ALT |  | 463(80.77) | 110(19.23) |
| TBIL | 5(0.92) | 535(98.16) | 15(0.92) |
| DBIL |  | 561(98.08) | 11(1.92) |
| UBIL |  | 558(97.55) | 14(2.45) |
| GLU | 42(7.50) | 325(58.04) | 193(34.46) |
| BUN | 1(0.18) | 43(7.53) | 527(92.29) |
| TC |  | 492(85.71) | 82(14.29) |
| TG |  | 365(63.59) | 209(36.41) |
| LDL-C |  | 541(94.25) | 33(5.75) |
| ApoB | 46(14.98) | 247(80.56) | 14(4.56) |
| PLIP | 153(33.19) | 292(64.34) | 17(3.47) |
| Cl^-^ | 102(17.83) | 382(66.78) | 88(5.39) |

FFAs, free fatty acids; ALT, alanine transaminase; AST, aspartate aminotransferase; TBIL, total bilirubin; DBIL, direct bilirubin; UBIL, unconjugated bilirubin; GLU, glucose; BUN, blood urea nitrogen; TC, total cholesterol; TG, triglyceride; LDL-C, low-density lipoprotein cholesterol; ApoB, apolipoprotein B; PLIP, phospholipid.


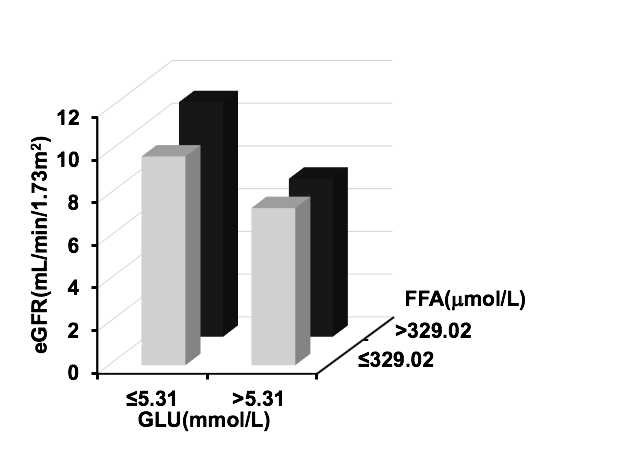
Figure S1. Serum FFAs and GLU exerted an opposite effect on renal function in CRF patients. CRF patients with higher serum GLU and lower serum FFAs showed an obvious decrease of eGFR. FFAs, free fatty acids; GLU, glucose, CRF, chronic renal failure.
